# Antiobesity Potential of Bioactive Constituents from Dichloromethane Extract of *Psoralea corylifolia* L. Seeds

**DOI:** 10.1155/2022/9504787

**Published:** 2022-08-25

**Authors:** Neha Mahajan, Bhupendra Koul, Jasleen Kaur, Mahendra Bishnoi, Pankaj Gupta, Amit Kumar, Bhahwal Ali Shah, Iqra Mubeen, Ashutosh Kumar Rai, Ram Prasad, Joginder Singh

**Affiliations:** ^1^Department of Biotechnology, Lovely Professional University, Phagwara, 144411 Punjab, India; ^2^Department of Biotechnology, Govt. Degree College Kathua, Affiliated to University of Jammu, 184104, J&K (UT), India; ^3^National Agri-Food Biotechnology Institute, Knowledge City-Sector 81, SAS, Nagar, Punjab 140603, India; ^4^Department of Chemistry, Govt. Degree College Kathua, Affiliated to University of Jammu, 184104, J&K (UT), India; ^5^CSIR-Indian Institute of Integrative Medicine, Canal Road, J&K (UT), Jammu 180001, India; ^6^College of Plant Health and Medicine, Key Lab of Integrated Crop Disease and Pest Management, Qingdao Agricultural University, Qingdao, Shandong 266109, China; ^7^Department of Biochemistry, College of Medicine, Imam Abdulrahman Bin Faisal University, Dammam 31441, Saudi Arabia; ^8^Department of Botany, Mahatma Gandhi Central University, Motihari, 845401 Bihar, India

## Abstract

**Purpose:**

Effectively controlling the accumulation of adipose tissue can be a therapeutic strategy for treating obesity, which is a global problem. The present study was designed for comparative assessment of *in vitro* antiobesity activities of the *Psoralea corylifolia*-dichloromethane seed extract (DCME) and the isolated phytochemicals, bakuchiol, isopsoralen, and psoralen, through antiadipogenesis and pancreatic lipase (PL) inhibition assays. *Material and Methods. In vitro* pancreatic lipase activity was determined spectrophotometrically by measuring the hydrolysis of *p*-nitrophenyl butyrate (*p*-NPB) to *p*-nitrophenol at 405 nm, and adipogenesis was assayed in 3 T3-L1 adipocytes (by using Oil Red O staining) using *P. corylifolia*-dichloromethane seed extract (DCME) and individual compounds, isolated from the extract.

**Result:**

Antilipase as well as antiadipogenesis activity was displayed by both the DCME and the compounds. Maximum antilipase property was recorded in DCME (26.02 ± .041%) at 100 *μ*g/ml, while, among the isolated compounds, bakuchiol exhibited a higher activity (24.2 ± 0.037%) at 100 *μ*g/ml concentration, compared to other isolates. DCME was found to exhibit antiadipogenesis property, 75 ± 0.003% lipid accumulation, compared to the control at 100 *μ*g/ml dose. Bakuchiol, isopsoralen, and psoralen inhibited the lipid accumulation in 3T3-L1 preadipocytes, 78.06 ± 0.002%, 80.91 ± 0.004%, and 80.91 ± 0.001%, respectively, lipid accumulation in comparison to control at 25 *μ*M dose.

**Conclusion:**

The present study highlights the antiobesity potential of *P. corylifolia* and its active constituents. Thus, it can be concluded that *P. corylifolia* has the potential to treat obesity and related diseases; however, further research on dose standardization and clinical trials are required.

## 1. Introduction

Obesity can be defined as the abnormal enhancement in the mass of adipose tissue in the body [1]. It is a complex multifactorial disease and a global health issue that leads to metabolic syndrome, diabetes, hypertension, heart failure, renal failure, and other comorbidities [2, 3]. When an adult's body mass index (BMI), a measurement of body fat based on height and weight, surpasses 25 or 30 kg/m^2^, they are called overweight or obese [4, 5]. Undoubtedly, obesity is caused by a sedentary lifestyle and the ingestion of extra calories on a regular basis. Overweight and obesity prevalence rates have roughly doubled globally since 1980, with over one-third of the world's population being categorized as overweight or obese [6].

Energy intake starts from fat absorption after digestion of fat into monoglycerides and fatty acids. Lipase is a crucial enzyme in the absorption of lipids [7]. The pancreatic lipase, which is responsible for the hydrolysis of 50-70% of total dietary fats, is known to facilitate fat digestion [8]. However, its suppression can lower the fat level in the blood and can be a useful strategy for the management of obesity [9]. Adipogenesis is a complex multistep process through which preadipocytes are converted into mature, lipid-containing adipocytes [10]. In obesity, adipocytes experience unusual augmentation characterized by enhanced numbers of fat cells storing their lipids through excessive adipocyte differentiation. So, disruption of adipocyte differentiation may decrease fat storage.

Although traditional therapies such as lifestyle modification (diet and exercise) and medication are useful, their weight loss results are limited. Despite the advances in our understanding of obesity, clinical therapy of the condition faces several challenges [11]. Patients and practitioners may be disappointed by the less favorable results of weight loss after consuming most of the pharmaceuticals, as well as weight recovery once they are discontinued. Hence, weight-loss medications are not a popularly used practice and may create some lingering doubts regarding obesity pharmacotherapy's long-term safety [12]. Currently, the US Food and Drug Administration has approved four weight-loss medicines: orlistat, marketed as Xenical® by Roche, Switzerland; Alli® by GlaxoSmithKline, UK; Contrave® by Nalpropion Pharmaceuticals, USA; Belviq® by Eisai, Japan; and Qsymia® by Vivus, USA. But existing synthetic medications have been linked to heart attacks, strokes, and liver damage [13].

Medicinal plants from any region serve as a natural reservoir for medications, and they are a boon as they can treat several ailments quickly and effectively [14]. Natural products have the advantage of providing complex phytochemical combinations with high structural complexity and biological potency [15]. Herbal treatments are independent of any age group or sex and help to stay healthy and active in the long run. In fact, they are the best remedy to decrease body fat and weight [16]. Drug discovery from medicinal plants led to the isolation of early drugs such as cocaine, codeine, digitoxin, and quinine, in addition to morphine, of which some are still in use.

*Psoralea corylifolia* L. (family: Fabaceae) is one such widely used medicinal plant whose extracts/compounds are reported to exhibit estrogenic, anticancer, antioxidant, antimicrobial, antidepressant, anti-inflammatory, osteoblastic, and hepatoprotective effects, among others [17]. The effect of *P. corylifolia* seed extract on high fat diet-induced nonalcoholic fatty liver disease (NAFLD) in C57BL/6 mice was studied, and it could decrease body weight as well as glucose level in blood. It also improved the glucose tolerance and insulin sensitivity [18]. Prenylated flavonoid-standardized extract (PFE) from the seeds of *P. corylifolia* was found to drastically reduce bodyweight, fat mass, and white adipose cells in the high fat diet-induced obese mice [19]. *P. corylifolia* is less explored plants on account of its antihyperlipidemic activity. Although there are several medicines in the market to treat obesity, prolonged intake can result in serious negative effects. As a result, scientists are on the lookout for novel natural sources with potential antiobesity activity. In order to find new bioactive natural antiobesity agents, compounds isolated from the *P. corylifolia* seed DCME were evaluated for their antilipase as well as antiadipogenesis activity. This is the first report wherein antiadipogenesis activity of the *P. corylifolia* DCME seed extract as well as of the isolated compounds has been reported.

## 2. Material and Methods

### 2.1. Plant Material

Seeds of *P. corylifolia* were procured from CSIR-IIIM Jammu and submitted to the Crude Drug Repository (CDR) of Janaki Ammal Herbarium at IIIM Jammu. CDR accession number allotted to the voucher specimen was CDR 4242. Dried seeds were grinded using a mechanical grinder, and the seed powder was sealed in a plastic bag and stored at room temperature (in a vacuum desiccator) until further use.

### 2.2. Chemicals and Reagents

Mouse 3T3-L1 preadipocytes were procured from the National Centre for Cell Science, Pune, India. Petroleum ether, dichloromethane, methanol, ethyl acetate, dimethyl-thiazol-diphenyl tetrazolium bromide, Dulbecco's modified eagle medium, dexamethasone, Bovine calf serum, penicillin, streptomycin, 3-isobutyl-1-methylxanthine, dimethyl sulfoxide (DMSO), Oil Red O, isopropyl alcohol, and formaldehyde were procured from Sigma Chemical Co. USA. All the chemicals utilized were of analytical grade.

### 2.3. Preparation of Plant Extracts

100 g of dried seeds were grinded, and then extracts were prepared by the maceration method. Three different solvents (petroleum ether, dichloromethane, and methanol) were used for sequential extraction starting from low polarity to high polarity [20, 21]. The same fresh solvent was used for two more extractions before switching to the next polar solvent. Plant extracts were evaporated to obtain the dry residue. The percentage yield of the dried extract was computed.

### 2.4. Ultrafast Liquid Chromatography (UFLC)

The analysis of DCME and the compounds isolated from the same extract was performed using Shimadzu UFLC system which was equipped with a gradient solvent quarternary pump (LC-20 AD), autosampler SIL-20A HT, column oven CTO-10ASvp, and detector PDA SPD-M20A. The analytes were separated by using column Merck RP-18e LiChrospher, having particle size 5 *μ*m. With gradient elution using acetonitrile (A) and 0.1 percent v/v formic acid in water (B) at a flow rate of 0.75 ml/min, the total run duration was 50 minutes. The gradient was as follows (in terms of v/v percent of B): 0−0.01 min, 90; 0.01-15 min, 90 to 70; 15-25 min, 70 to 40; 25-35 min, 40 to 0; 35-40 min, 0; 40-45, 0 to 90; and 45-50 min, 90.

### 2.5. Isolation and Characterization

For isolating the secondary metabolites from *P. corylifolia* seeds, silica gel column chromatography technique was applied to the DCME. A vertical glass column (40 mm wide by 60 mm long) made of borosilicate material was used for the fractionation. The column was rinsed well with acetone and was completely dried before packing. A piece of glass wool was placed at the bottom of the column with the help of a glass rod. Silica slurry was prepared with hexane and was poured from the top of the column approximately 2/3rd of the column. The extract (9.46 g) was loaded onto column packed with 60-120 mesh size silica gel and gradually eluted with 1 to 15% (vol/vol) ethyl acetate (EtOAc) in n-hexane. Each chromatographic fraction which was collected was 15-20 ml and was examined by TLC using TLC silica gel 60 F_254_ sheets (Merck). The separated compound on TLC was detected under the UV Fluorescence Analyzing Cabinet (Sunstar). After the evaluation of the TLC, similar *R*_*f*_ value fractions were pooled and allowed to evaporate. Analysis of ^1^H NMR (400 MHz) and ^13^C NMR (125 MHz) spectra was done with Bruker FT-NMR spectrometer and compared with the data published [22] which allowed to predict the structure of the isolated compounds.

### 2.6. Pancreatic Lipase Inhibition Assay

The pancreatic lipase inhibition assay was performed according to the procedure by Sridhar et al. [23] with slight modification. The enzyme solution was prepared immediately before use, and for this, 25 mg of porcine pancreatic lipase was suspended in 5 ml of the TrisHCl buffer (pH 7.4). The solution was then subjected to centrifugation (4000 rpm, 18°C for 10 min). The supernatant was collected and used. For all, i.e., DCME, the isolated compounds and the positive control (orlistat), different concentrations (100 *μ*g/ml, 50 *μ*g/ml, and 25 *μ*g/ml) were prepared in TrisHCl buffer containing 1% dimethyl sulfoxide. The final 1000 *μ*l reaction mixture is comprised of a preincubated mixture (5 min at 37°C) of 875 *μ*l of buffer, 100 *μ*l of enzyme, and 20 *μ*l of DCME/isolated compounds/orlistat of different concentrations, followed by the addition of 5 *μ*l of the substrate (4-nitrophenyl butyrate, 10 mM in acetonitrile).

The absorbance of the final mixture was determined using the microplate spectrophotometric reader (Spectra Max ABS Plus, USA) after 5 min at 405 nm. The assay was performed in triplicate, and the percentage inhibition was calculated using the formula

(1) $$\begin{array}{c} \%\text{Inhibition}=\left[\frac{\left(\text{AE}-\text{AT}\right)}{\text{AE}}\right]\times 100, \end{array}$$

where AE is the absorbance of enzyme control (without inhibitor) and AT is the difference between the absorbance of test sample, with and without substrate.

### 2.7. Cell Viability Assay

An MTT (dimethyl-thiazol-diphenyl tetrazolium bromide) assay was done in order to find out the cytotoxic effect of DCME/isolated compounds on the 3T3L1 cell line [24]. Undifferentiated 3T3L1 cells were seeded in a 96-well culture plate, and the density was kept at 1 × 10^4^ cells/ml. After 24 h, the medium was replaced with a medium having DCME or isolates at different concentrations. Incubation was done in a 5% CO_2_ atmosphere at 37°C for 24 h. The test solutions in the wells were removed after incubation, and 10 *μ*l of MTT (5 mg/ml MTT in PBS (pH = 7.4)) was added to each well. Thereafter, the plates were incubated at 37°C for 4 h. The supernatant was withdrawn, and 100 *μ*l of DMSO was added to the plates and agitated to dissolve the formazan that had formed. The absorbance was read at 570 nm using a microplate reader (SpectraMax i3 Molecular Devices, USA). Undifferentiated preadipocytes treated with 1% DMSO were used as control. The growth inhibition percentage was computed.

(2) $$\begin{array}{c} \%\text{Inhibition}=\left(\frac{\text{Mean test OD}}{\text{Mean non}‐\text{treated OD}}\right)\times 100. \end{array}$$

### 2.8. Cell Culture and Antiadipogenesis Assay

Mouse 3T3-L1 preadipocytes were cultured in a 250-ml culture flask with Dulbecco's Modified Eagle Medium (DMEM) supplemented with 10% Bovine Calf Serum (BCS) and 1% penicillin-streptomycin. Whole cell culture was maintained at 37° C in a humidified incubator along with a continuous supply of 95% O_2_ and 5% CO_2_ [25]. Postconfluent cells (80% confluency) were then stimulated to differentiate by incubating for 48 h in the medium containing basal medium (DMEM) with 10% BCS, 1% penicillin-streptomycin solution, and IDM cocktail (1 *μ*g/ml insulin, 0.1 *μ*M dexamethasone, and 0.1 mM 3-isobutyl-1-methylxanthine (IBMX)). The medium was replaced after two days with maintenance medium containing DMEM supplemented with 10% BCS, 1% penicillin-streptomycin solution, and 1 *μ*g/ml insulin for the next ten days, with medium replacement on every alternate day. Treatment of various compounds was given in differentiation medium as well as in maintenance medium for 12 days total. The control group was the cell lines treated with differentiated medium plus maintenance medium.

### 2.9. Oil Red “O” Staining

Lipid accumulation was measured using a method previously described [26]. During adipogenesis experiment, 3T3-L1 cells were treated with plant extracts/compounds. Cells were rinsed in PBS and then fixed with 3% formaldehyde in PBS for 30 minutes at room temperature before being incubated with oil red “O” solution (0.5 percent oil red “O” dye in isopropanol: water; 60 : 40). The excess dye solution was withdrawn, and the cells were rinsed with water to eliminate any remaining dye. An inverted microscope was used to capture images of the treated cells (Leica DMI 6000 B, Germany). After air drying the stained cells overnight, bounded dye separation in isopropanol was performed, and absorbance was read at 520 nm using an ELISA plate reader (SpectraMax i3 Molecular Devices, USA).

### 2.10. Statistical Analysis

The data was reported as mean ± SEM. GraphPad Prism software was used to analyze the significant differences between the means of different groups through one-way analysis of variance (ANOVA). This was followed by Tukey's post hoc test for comparison of means. For all results, *p* < 0.05 was considered statistically significant.

## 3. Results

### 3.1. Determination of Extractive Value of *P. corylifolia*

The dry powdered plant material of *P. corylifolia* (seeds) was extracted with solvents of different polarity, viz. petroleum ether, dichloromethane, and methanol using a maceration process. In a weighing bottle, 100 g of coarsely powdered plant material (seeds) was weighed and put to a dry 1-liter conical flask. Thereafter, petroleum ether was poured into the flask (500 ml). The flask was corked and incubated at room temperature for 24 h, with intermittent shaking. The liquid was filtered into a 500-ml beaker using a Whatman No. 1 filter paper. The residue was collected, dried, and transferred to a dry 1-liter flask, which was then filled with 500 ml dichloromethane and stirred for 24 h at 120 rpm at 37° C. The liquid was filtered into a 500-ml beaker using Whatman No. 1 filter paper. The residue was collected, dried, and transferred to a dry 1-liter flask, which was then filled with 500 ml of methanol, corked, and stirred for 24 h. The liquid was filtered into a 500-ml beaker using Whatman No. 1 filter paper. The same fresh solvent was used for two extractions before switching to the next polar solvent. To obtain crude extracts, all the solvents were evaporated under vacuum. When compared to other extracts, studies found that DCME had the highest extractive value.

### 3.2. Isolation and Biochemical Characterization of Phytochemicals

**Figure 1:**
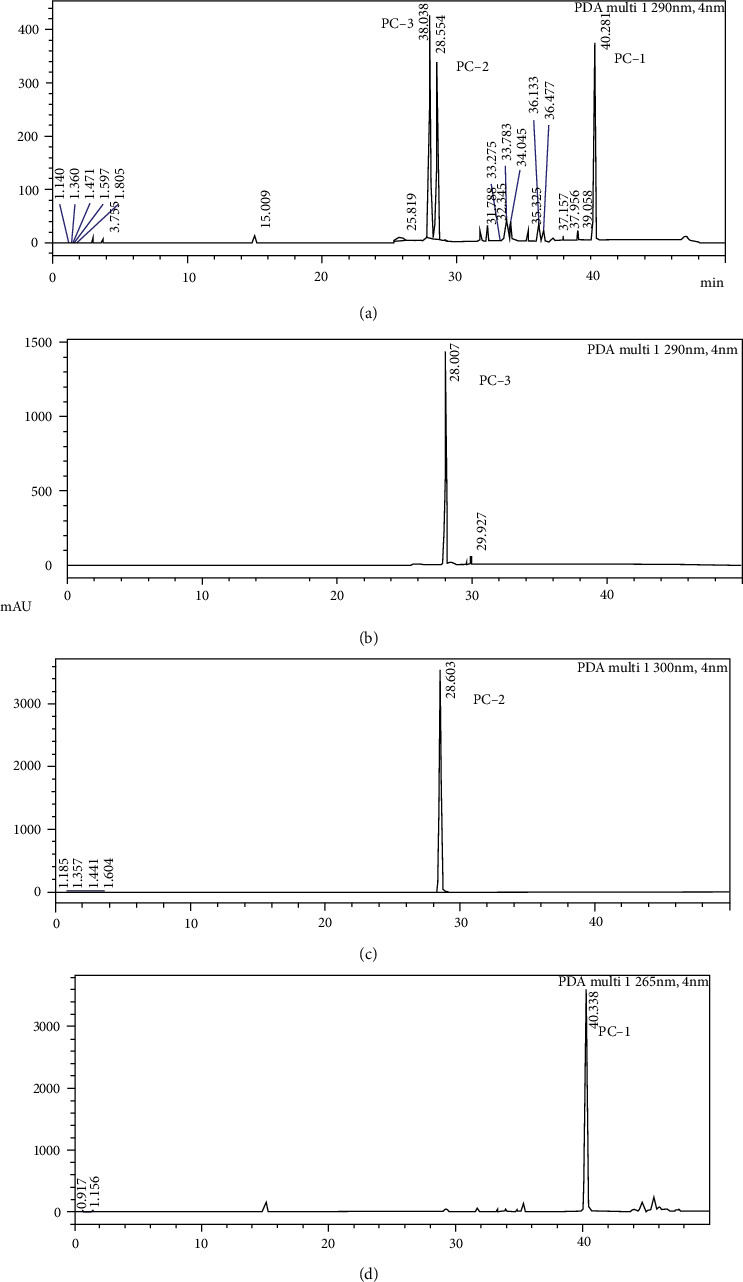
Ultrafast liquid chromatography (UFLC) chromatograms of *Psoralea corylifolia* seed extract and isolates. (a) Chromatogram of dichloromethane extract DCME, (b) chromatogram of PC-3 isolated by column chromatography from DCME, (c) chromatogram of PC-2 isolated by column chromatography from DCME, and (d) chromatogram of PC-1 isolated by column chromatography from DCME.

The DCME of *P. corylifolia* seeds yielded three major constituents. Using the slurry pack method with 100% hexane, 9.6 g of crude extract was put into the column. The sample was then gradually eluted in n-hexane with 1 to 15% (vol/vol) ethyl acetate (EtOAc). Each chromatographic fraction was then examined by TLC. Compound 1 having *R*_*f*_ value of 0.65 was obtained from fractions 7 to 19 [up to 5% (vol/vol) ethyl acetate in n-hexane]. TLC examination of the fraction was performed in a solvent ratio of 20% ethyl acetate in hexane. The combined organic solvent from different fractions was evaporated under vacuum to obtain compound 1 (0.6 g) as brown oily. The UFLC analysis of compound obtained above (compound 1) was carried out, and the chromatogram (Figure 1(d)) showed one single peak at the retention time of 40.3 min. This suggests that compound 1 is a pure molecule, and the structural elucidation using NMR spectra indicates that compound 1 is “bakuchiol.”

Compound 2 was obtained from the later obtained fractions 22-34 [solvent system up to 13% (vol/vol) ethyl acetate in n-hexane) of DCME which were then combined. The TLC analysis of the combined fractions was performed in a solvent system containing 20% ethyl acetate in hexane, after which the result was observed under UV light, and the *R*_*f*_ value of compound 2 was found to be 0.45. The combined organic solvent from different fractions was evaporated under vacuum to obtain compound 2 (0.09 g). The UFLC analysis of compound obtained above was carried out, and the chromatogram showed one single peak at the retention time of 28.5 min. Compound 2 was obtained as a white crystalline solid having melting point of 137-138°C.

Characteristic absorption as well as splitting pattern in 1H-NMR of compound 2 are in consonance with the 1H-NMR absorption and splitting pattern already reported by Jiangning et al. [22]. NMR spectra indicate that compound 2 is “isopsoralen.”

Compound 3 (40 mg) was isolated from subfraction obtained by combining fractions 35-45, which was subjected to repeated CC using gradient elution with 0-15% ethyl acetate in n-hexane as analyzed by TLC. The *R*_*f*_ value of compound 3 was found to be 0.31. The combined organic solvent was evaporated under vacuum to obtain compound 3 as white solid (0.04 g) having melting point 163-164°C. The UFLC analysis of compound 3 was carried out, and the chromatogram (Figure 1(b)) showed one single peak at the retention time of 28.0 min. NMR spectra studies indicate that compound 3 is “psoralen.” After isolation of three major chemical constituents from DCME, our next aim was to study the antiobesity potential of these components.

### 3.3. Antilipase Activity of DCME and Isolated Compounds of *P. corylifolia*

**Figure 2:**
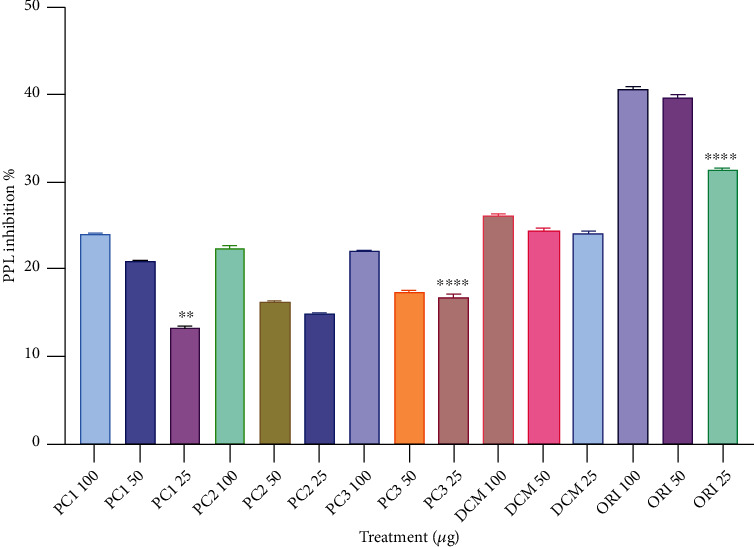
Percentage inhibition of pancreatic lipase enzyme by DCM extracts and isolated compounds. Each value is expressed as a mean ± SEM. Each experiment was performed in triplicate (^∗^*p* < 0.05, ^∗∗^*p* < 0.01^∗∗∗^ <0.001). Results revealed that all the isolates as well as extract exhibited a dose-dependent inhibitory activity against pancreatic lipase.

Lipids are an essential component of human nutrition. Inhibiting the digestion of dietary fats can be a rational target for pharmaceutical intervention [27]. The antilipase activity of the DCME of *P. corylifolia* seeds, bakuchiol (PC1), isopsoralen (PC2), and psoralen (PC3) was performed at 100 *μ*g/ml, 50 *μ*g/ml, and 25 *μ*g/ml concentration. The percentage inhibition of PL enzyme recorded in each experiment is shown in Figure 2.

### 3.4. MTT Assay

Before testing the extracts/compounds with the 3 T3-L1 cell line, a toxicity test was required to determine the concentration at which the extract becomes toxic, in order to rule out the possibility that the extract does not influence cell viability. The required level of viability should be greater than 90% [28].

**Figure 3:**
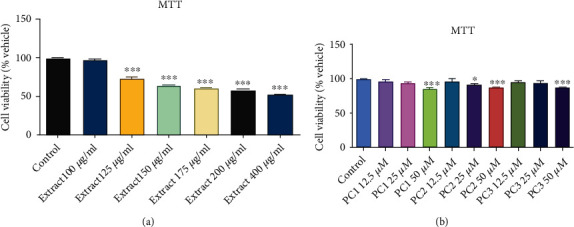
Viability of 3 T3-L1 adipocytes under various concentrations of (a) DCM extract of *P. corylifolia* seeds and (b) isolated compounds PC1 (bakuchiol), PC2 (isopsoralen), and PC3 (psoralen) as determined by MTT assay. Data are expressed as mean ± SEM (^∗^*p* < 0.05, ^∗∗^*p* < 0.01^∗∗∗^ <0.001 as compared to control).

For toxicity analysis, 3 T3-L1 cells were treated with DCME at different concentrations 100, 125, 150, 175, 200, and 400 *μ*g/ml. At concentrations up to 100 *μ*g/ml, DCME of *P. corylifolia* showed no substantial toxicity to 3 T3 -L1 cells, as shown in Figure 3(a). The viability of 3T3-L1 cells was severely reduced at concentrations more than 100 *μ*g/ml. For further tests, safe doses of DCME were kept 100 *μ*g/ml.

The MTT assay was also used to determine the effect of bakuchiol, isopsoralen, and psoralen from *P. corylifolia* seed on 3T3-L1 cell viability and the cells were treated with different concentrations of pure compounds, i.e., 12.5, 25, and 50 *μ*M. At concentrations up to 25 *μ*M, none of the isolates were found to be toxic to 3T3 -L1 cells. The viability of 3T3-L1 cells was reduced at concentrations greater than 25 *μ*M (Figure 3(b)). Thus, safe doses of PC-1, PC-2, and PC-3 were determined as up to 25 *μ*M for further experiments.

### 3.5. Antiadipogenesis Assay

The differentiation of preadipocytes to mature adipocytes is connected with the increase in the number of cells stained with Oil Red O and lipid accumulation. 3T3-L1 cells were treated with differentiation media plus 100 *μ*g/ml of DCM extract or 25 *μ*M of bakuchiol/isopsoralen/psoralen for 48 hours to see if *P. corylifolia* influences adipogenesis. Further, the cells were shifted to maintenance medium (basal medium with 1 *μ*g/ml insulin) for 10 days with medium replacement on every alternate day. For the antiadipogenesis experiment, extracts or isolates were introduced to differentiation and maintenance medium in 0.1% DMSO carrier for up to 12 days. Control is cells treated with differentiation media plus maintenance media.

**Figure 4:**
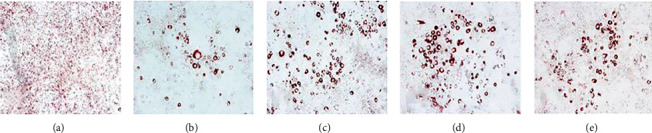
Images of treated cells obtained using an inverted microscope. (a) Control, (b) PC1 treated, (c) PC2 treated, (d) PC3 treated, and (e) DCM extract treated. Red spots in images represent area stained by Oil Red O Dye.

**Figure 5:**
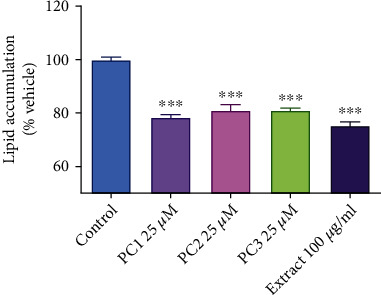
Inhibition of fat accumulation in 3T3-L1 via suppression of adipogenesis by DCM extract/isolated compounds from *Psoralea corylifolia* as determined by Oil O Red solvent extraction. Data are expressed as mean ± SEM (*n* = 5). (∗ <0.05, ∗∗ <0.01, and ∗∗∗ <0.001 versus the control group). The control group was the cell lines treated with differentiated medium plus maintenance and was considered as 100% lipid accumulation.

After staining with Oil Red O stain on day 12th, cells were imaged using an inverted microscope. The Oil Red O stain demonstrates a reduction in the quantity and size of lipid droplets accumulated in the cells treated with extract or isolates, when viewed under microscope. DCM extract as well as bioactive compounds treatment led to inhibition of adipogenesis (Figures 3(a)–3(e)). After capturing images, OD detection was performed. The results indicated that treatment with the extract or compounds led to a significant decrease of OD (Figure 4). Specifically, treatment with DCM extract, bakuchiol, isopsoralen, and psoralen led lipid accumulation to 75 ± 0.003%, 78.06 ± 0.002%, 80.91 ± 0.004%, and 80.91 ± 0.001% in comparison to control, respectively (Figure 5).

## 4. Discussions

Obesity is currently one of the main public health concerns since it is a major contributor to the global burden of chronic diseases, including cardiovascular disease, nonalcoholic fatty liver disease, type 2 diabetes mellitus, and certain types of cancer. Obesity-associated comorbidities can impact function and quality of life as well as mental health also [29]. Phytochemicals are plant-based chemicals that have been shown to be useful in the treatment of chronic diseases like cancer, diabetes, and obesity. Polyphenols and flavonoids have been shown to be particularly abundant in extracts that may be useful against diseases [30].

Phytochemical studies indicated that coumarins, flavonoids, and meroterpenes are the main components of *P. corylifolia.* Some of its isolated compounds are bakuchiol, psoralidin, psoralen, isopsoralen, bavadin, isobavachalcone, corylin, daidzin, and genistein. The extracts and active components of *P. corylifolia* demonstrated multiple biological activities, including estrogenic, antitumor, antioxidant, antimicrobial, antidepressant, anti-inflammatory, osteoblastic, hepatoprotective [17], and antiobesity activities [31]. Due to the various biological activities of this plant, much interest has been generated, suggesting further study on its effect on antiobesity. Polyphenols, flavonoids, terpenoids, alkaloids, saponins, carboxylic acids, glycosides, and tannins are some of the natural secondary metabolites found in plants that have been reported to have antiobesity properties through various modes of action [32]. In the present study, we found that the isolated phytochemicals from *P. corylifolia* possess antiobesity activity, and bakuchiol is a type of meroterpenoid, and psoralen and isopsoralen are coumarins (phenolic substance). Our data supports the potential of terpenoids and phenols as antiobesity agents. Moreover, in a study, PCS extract treatment significantly attenuated lipid accumulation in liver and adipose tissue and reduced serum lipid and hepatic triglyceride levels [18]. So, this study favors our work to choose *P. corylifolia* for antiobesity activity.

One of the most extensively utilized models for examining the potential efficacy of natural products as antiobesity medicines is pancreatic lipase inhibition [33]. Since inhibitors of digestive lipase should be useful antiobesity agents, recent research has focused on identifying novel and safe lipase inhibitors from natural sources like phytic acid, tannin, saponins, and Oolong tea [34]. In our study, by assessing the hydrolysis of p-NPB to p-nitrophenol, an in vitro pancreatic lipase model was utilized to assess the inhibitory effect on pancreatic lipase of all isolates as well as of DCME of *P. corylifolia* at concentrations of 25, 50, and 100 *μ*g/ml. The results from our study indicated that DCM extract of *P. corylifolia* seeds, bakuchiol, isopsoralen, and psoralen exhibited inhibitory activity against pancreatic lipase which is consistent with the activity reported by other groups [35]. For comparison the antilipase activity of orlistat (positive control) was also studied which was found to be more active than our isolated compounds. Noteworthy, weaker inhibitory effect of plant extracts and their isolated compounds on pancreatic lipase than orlistat has been observed by others [36]. Weaker antilipase effect than orlistat may be ascribed to the complexity of composition and multiple interactions between different components. Although orlistat is an available market drug, but some of its most common side effects (gastrointestinal toxicity, liver damage, pancreatic damage, kidney damage, metabolic system abnormalities, and a high cancer risk) severely limit its therapeutic applicability, particularly for long-term use [37].

When energy intake exceeds energy expenditure over time, the surplus energy is stored in the form of triglycerides in adipose tissue. An increase in cell size, cell number, or both can result in increased adipose tissue mass [38]. Preadipocyte differentiation has become an area of intense research in recent years with in vitro models of adipogenesis, such as the 3T3-L1 cell line, being used to investigate it [39]. Lipid droplet accumulation and preadipocytes differentiation into mature adipocytes are regarded as the hallmark events in obesity [40]. We examined the effects of the compounds on the inhibition of lipid accumulation after conducting the MTT assay. In the present study, as detected by the Oil Red O staining, adipogenesis and lipid accumulation were markedly inhibited by treatment with DCME of *P. corylifolia* seeds, bakuchiol, isopsoralen, and psoralen in 3T3-L1 adipocytes. It is interesting to note that DCME displayed a higher activity compared to the isolated compounds. This could be due the cumulative effect of other known compounds such as genistein [41, 42], bavachin [43], isobavachalcone [44], and corylin [45] in the *P. corylifolia* seed extract which are known to possess antiobesity potential. There are several reports which revealed the inhibition of adipogenesis via regulation of PPAR*γ* and C/EBP*α* expression in 3T3-L1 cells [46]. *P. corylifolia* may inhibit adipocyte differentiation by suppressing PPAR*γ* and C/EBP*α* transcriptional activity in 3T3-L1 adipocytes.

## 5. Conclusions

Through our studies on PCSE, we claim that *P. corylifolia* possesses antiobesity potential, but there are only few scientific evidences to support this claim [31]. The antiobesity activity of *P. corylifolia* seed extract might be due to the presence of coumarins [47], flavonoids [48], etc. Present work disclosed that bakuchiol, isopsoralen, and psoralen isolated from PCSE inhibited pancreatic lipase 24.2 ± 0.037, 22.69 ± 0.026, and 22.24 ± 0.057 percent, respectively, at 100 *μ*g/ml concentration. Bakuchiol, isopsoralen, and psoralen decreased lipid accumulation in 3T3-L1 preadipocytes with 78.06 ± 0.002%, 80.91 ± 0.004%, and 80.91 ± 0.001% lipid accumulation, respectively, in comparison to control at 25 *μ*M dose. These findings indicate the involvement of phytochemicals derived from *P. corylifolia* in regulating adipocyte differentiation, making it a promising candidate for the treatment of obesity-related diseases. This work highlights the therapeutic role of PCSE in metabolic disorders, which can help researchers across globe to analyze the various constituents responsible for antiobesity activity on specific receptors in adipose tissue. Thus, extensive research could be planned to determine the precise mode of action of the aforementioned bioactive compounds.

## Data Availability

The data used to support the findings of this study are included within the article.

## Additional Points

*Highlights*. (i) Bioactive constituents (bakuchiol, isopsoralen, and psoralen) were isolated from *Psoralea corylifolia*-dichloromethane seed extract. (ii) *Psoralea corylifolia*-dichloromethane seed extract exhibited antiadipogenesis and pancreatic lipase activities. (iii) Seed extract and isolated compounds showed therapeutic potential against adipogenesis

## Supplementary Materials

Supplementary Materials Supplementary Table 1 The extractive value of different extracts. Supplementary Table 2: spectral and melting point data of the compounds isolated from *Psoralea corylifolia* L. Supplementary Figure 1: 1H NMR and 13C NMR of different isolates, isolated by column chromatography, from DCM extract of *Psoralea corylifolia*. (a) 1H NMR of PC-1, (b) 13C NMR of PC-1, (c) 1H NMR of PC-II, (d) 13C NMR of PC-II, (e) 1H NMR of PC-III, and (f) 13C NMR of PC-III.
