# Supplementary material for: Antiobesity Potential of Bioactive Constituents from Dichloromethane Extract of Psoralea corylifolia L. Seeds
Source: Biomed Res Int. 2022 Aug 25;2022:9504787. doi: 10.1155/2022/9504787 (PMC9436577; doi:10.1155/2022/9504787)
Supplement: Supplementary Materials — Supplementary Table 1 The extractive value of different extracts. Supplementary Table 2: spectral and melting point data of the compounds isolated from Psoralea corylifolia L. Supplementary Figure 1: 1H NMR and 13C NMR of different isolates, isolated by column chromatography, from DCM extract of Psoralea corylifolia. (a) 1H NMR of PC-1, (b) 13C NMR of PC-1, (c) 1H NMR of PC-II, (d) 13C NMR of PC-II, (e) 1H NMR of PC-III, and (f) 13C NMR of PC-III. [file 9504787.f1.zip › Supplementary Figure.docx]

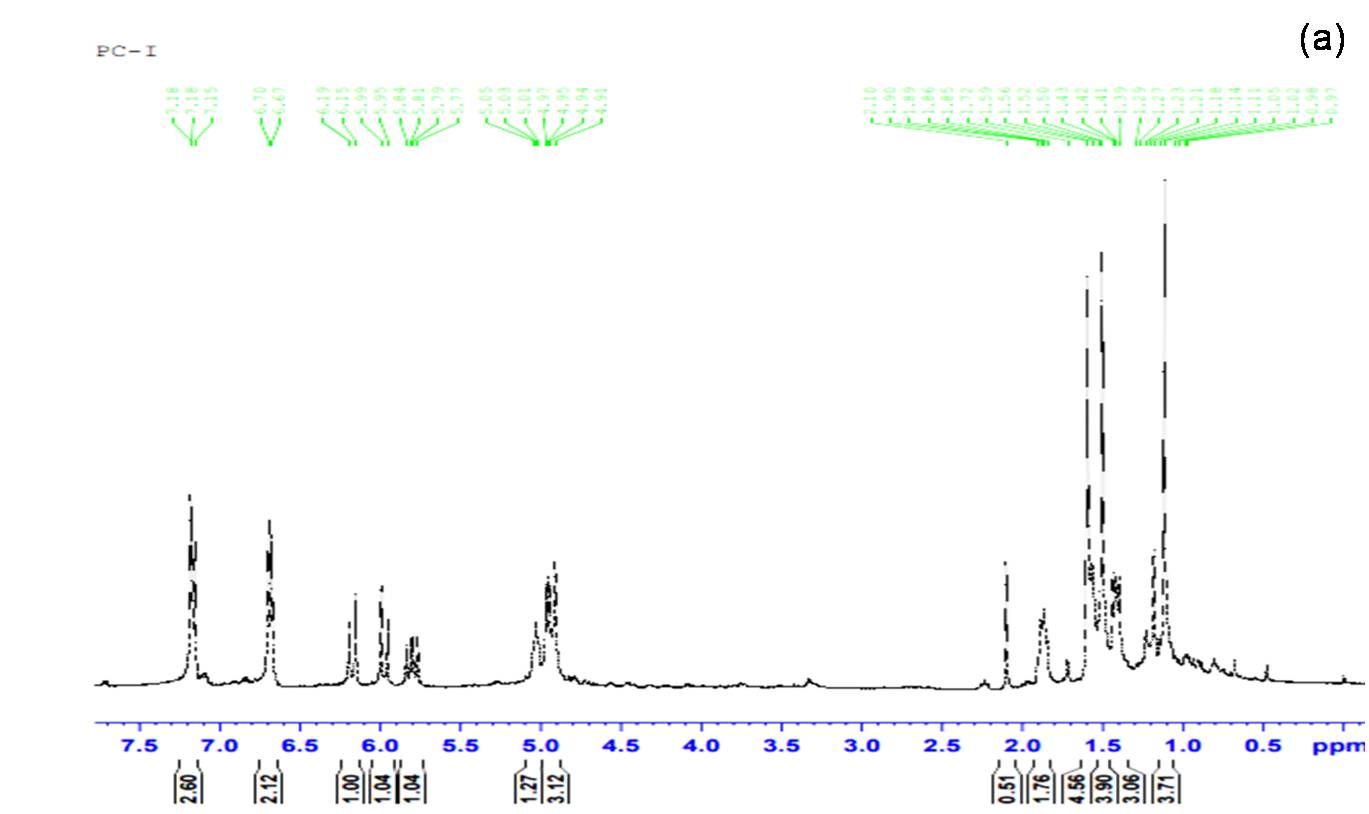


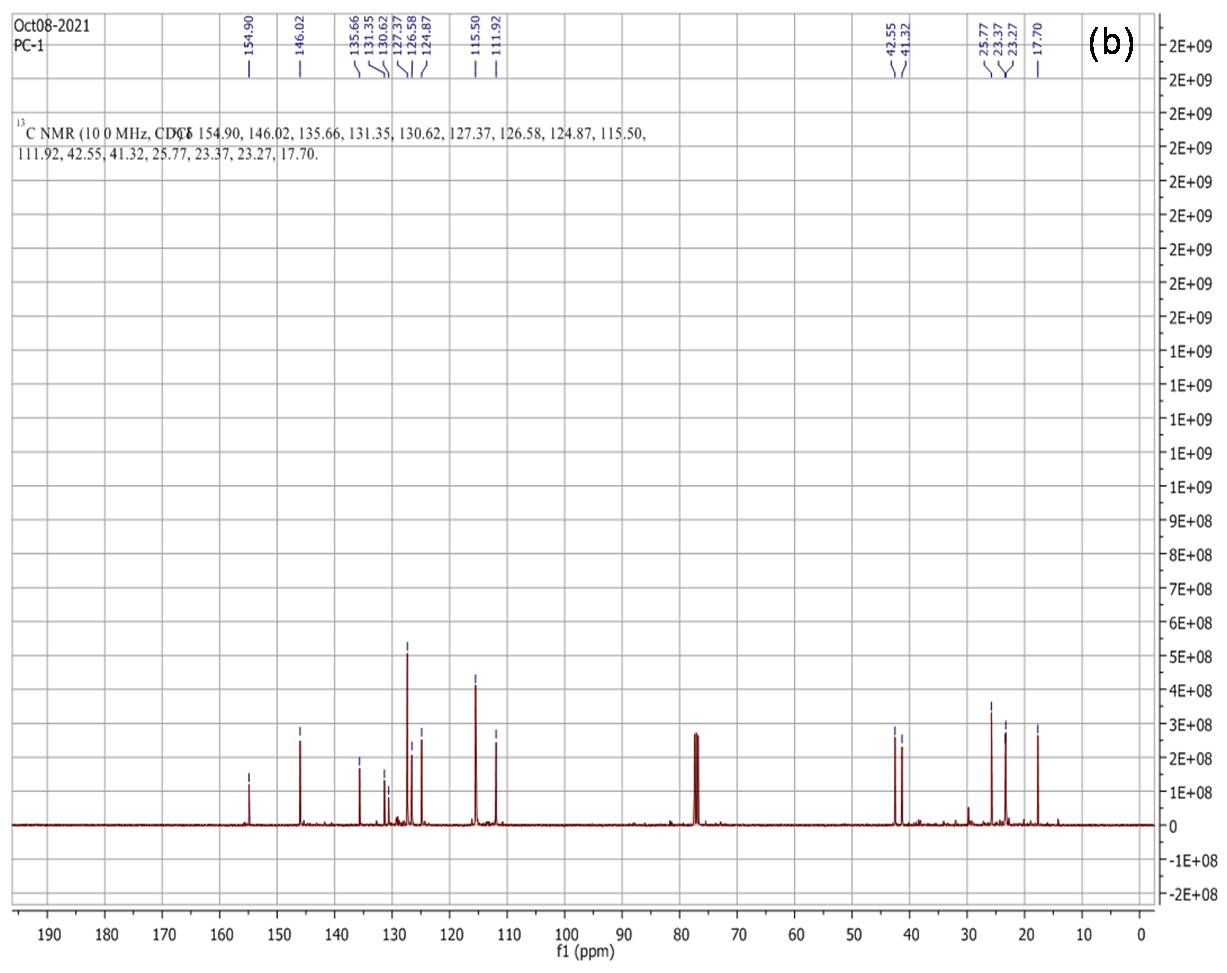


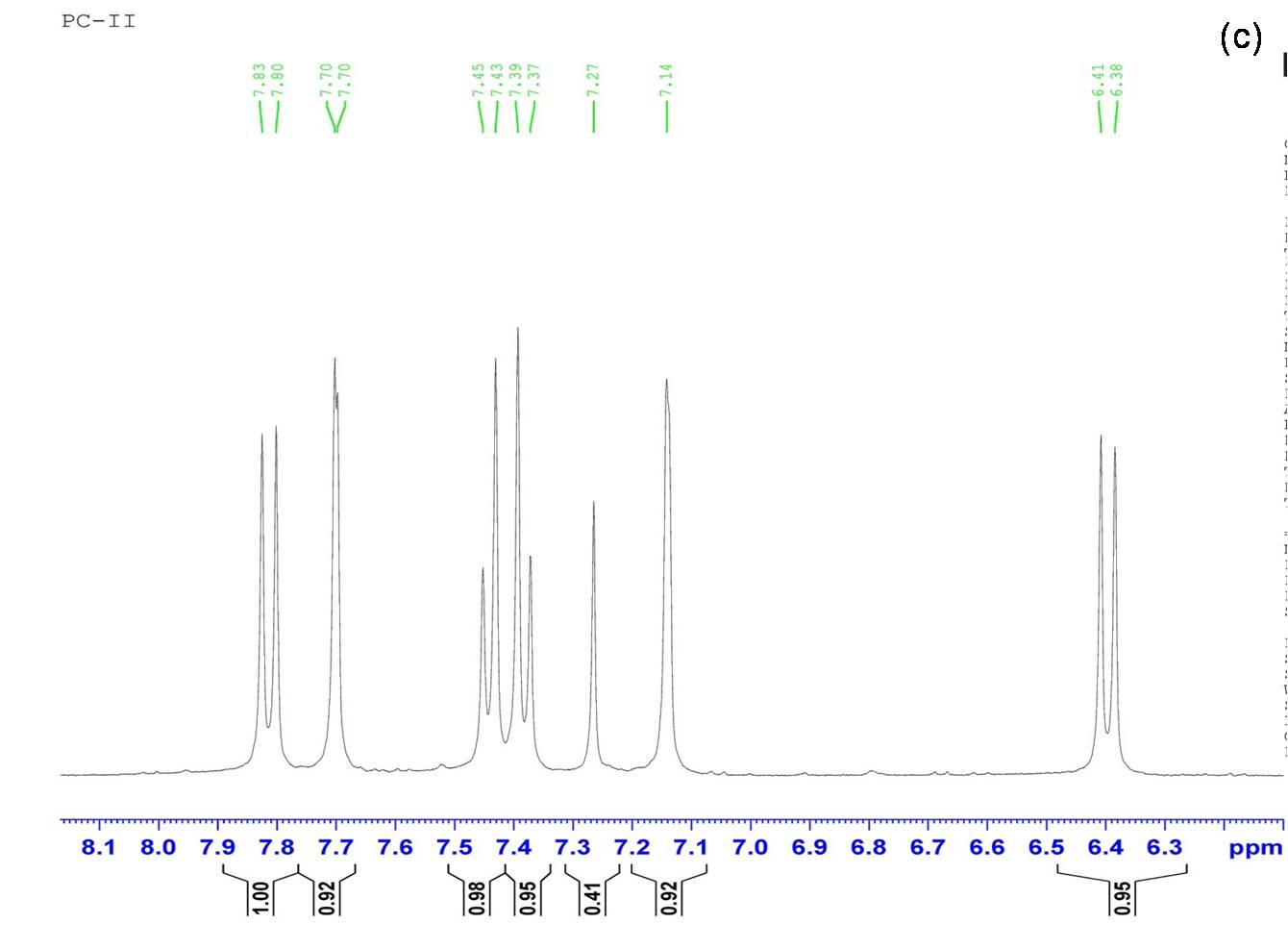


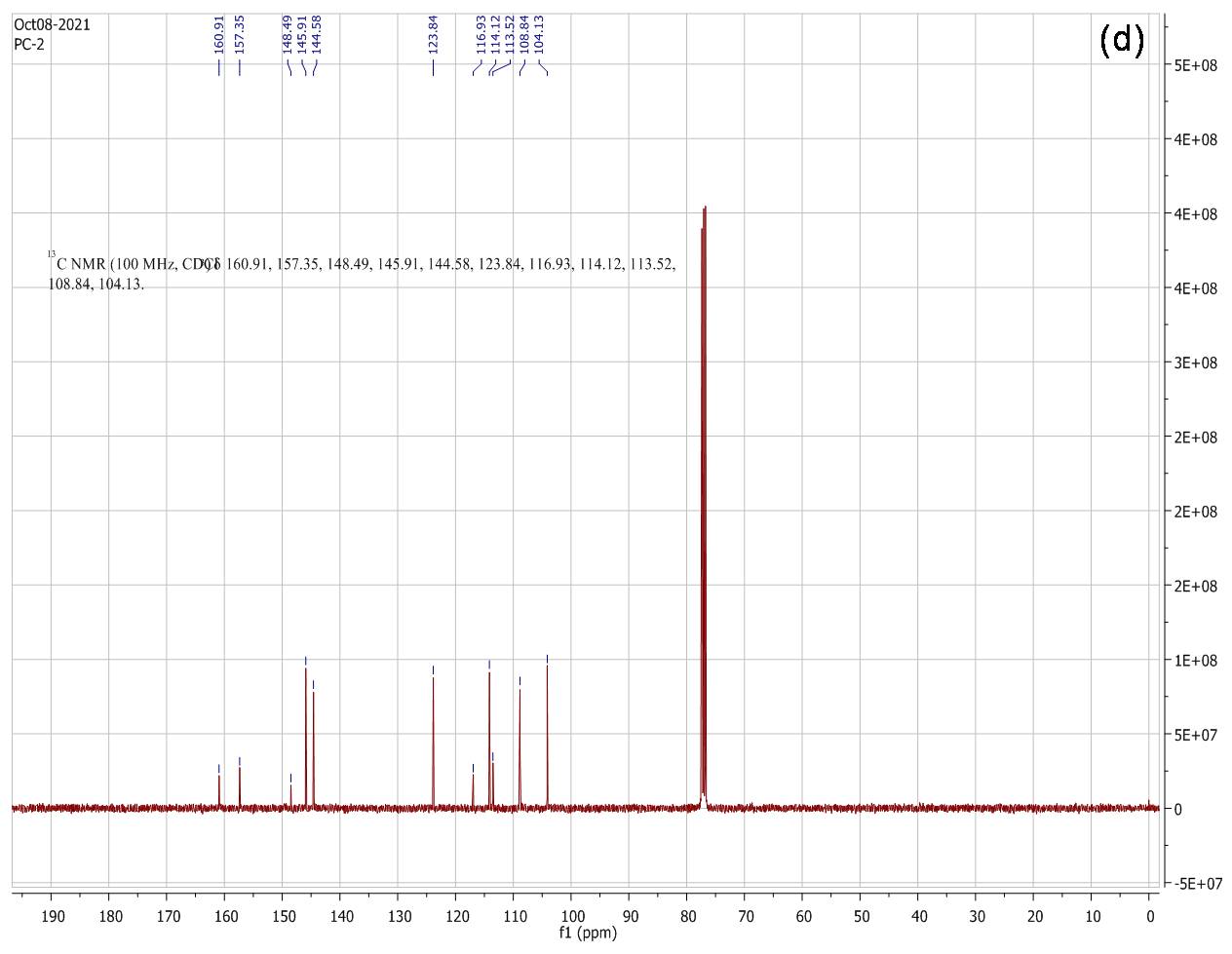


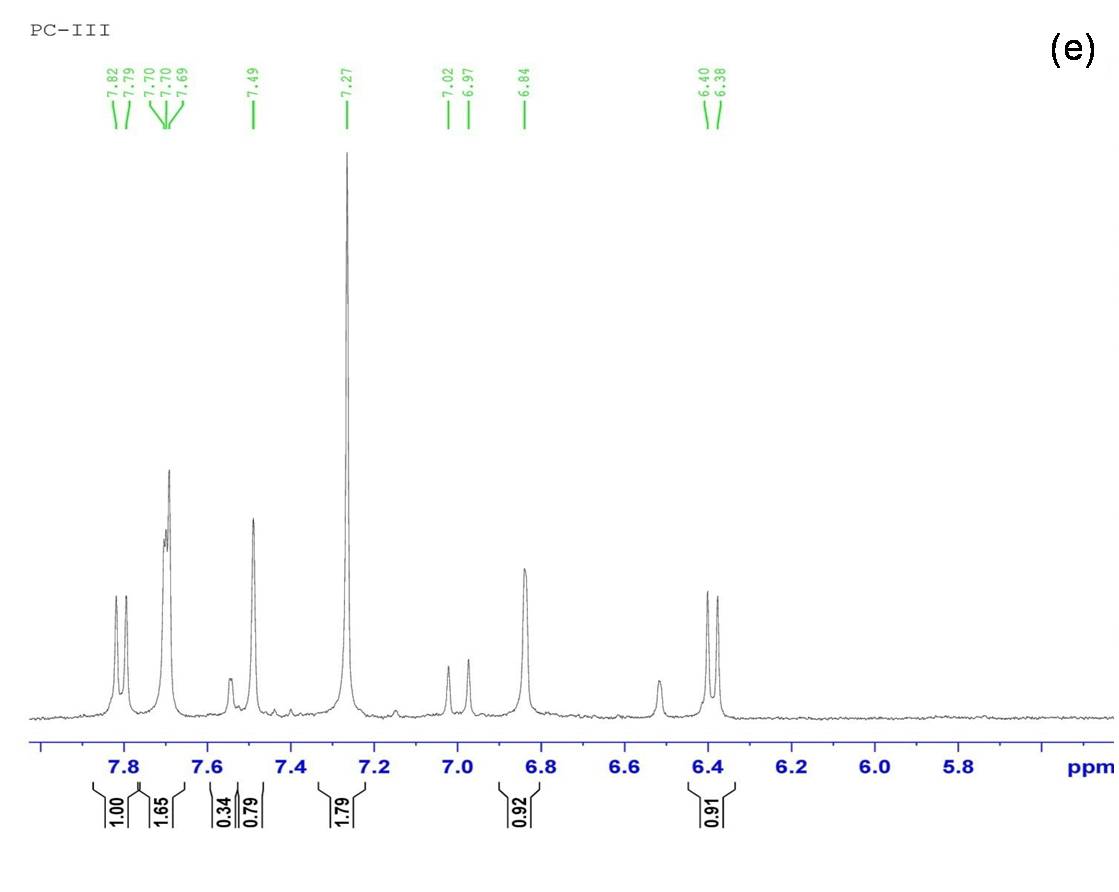


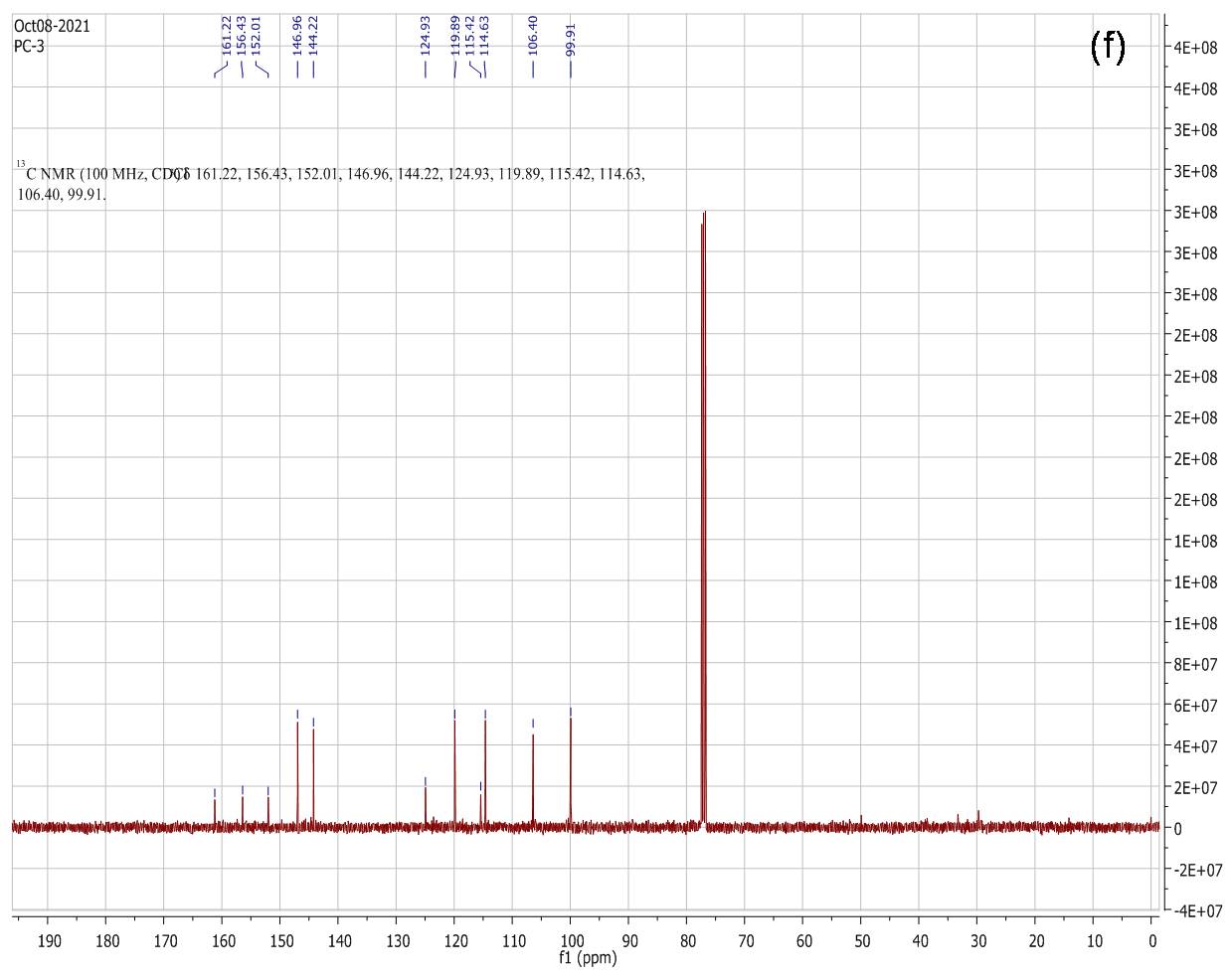


**Supplementary Figure 1** ^1^H NMR and ^13^C NMR of different isolates, isolated by column chromatography, from DCM extract of *Psoralea corylifolia.* (a) ^1^H NMR of PC-1 (b) ^13^C NMR of PC-1 (c) ^1^H NMR of PC-II (d) ^13^C NMR of PC-II (e) ^1^H NMR of PC-III (f) ^13^C NMR of PC-III
