# Supplementary material for: Antiobesity Potential of Bioactive Constituents from Dichloromethane Extract of Psoralea corylifolia L. Seeds
Source: Biomed Res Int. 2022 Aug 25;2022:9504787. doi: 10.1155/2022/9504787 (PMC9436577; doi:10.1155/2022/9504787)
Supplement: Supplementary Materials — Supplementary Table 1 The extractive value of different extracts. Supplementary Table 2: spectral and melting point data of the compounds isolated from Psoralea corylifolia L. Supplementary Figure 1: 1H NMR and 13C NMR of different isolates, isolated by column chromatography, from DCM extract of Psoralea corylifolia. (a) 1H NMR of PC-1, (b) 13C NMR of PC-1, (c) 1H NMR of PC-II, (d) 13C NMR of PC-II, (e) 1H NMR of PC-III, and (f) 13C NMR of PC-III. [file 9504787.f1.zip › Supplementary tables.doc]

**Supplementary Table 1.** The extractive value of different extracts

| **S. No.** | ***Psoralea corylifolia* extract** | **Weight of the extract (g)** | **Extractive value (%)** |
| --- | --- | --- | --- |
| 1 | Petroleum ether extract (PEE) | 5.29 | 5.29 |
| 2 | Dichloromethane extract (DCME) | 9.64 | 9.64 |
| 3 | Methanol extract (ME) | 7.62 | 7.62 |

**Supplementary Table 2.** Spectral and melting point data of the compounds isolated from *Psoralea corylifolia* L

| **Compound** | **Data of spectra** | | **Nature & MP (^o^C)** | **Literature Values (Jiangning et al 2005)** | |
| --- | --- | --- | --- | --- | --- |
|  | **^1^H NMR (400 MHz)**  **TMS as int. standard** | **^13^C NMR (100 MHz, CDCl3)** δ:  **TMS as int. standard** |  | **^1^H NMR (400 MHz)**  **TMS as int. standard** | **^13^C NMR (400 Hz)**  **TMS as int. standard** |
| Bakuchiol | 1.14 (s, 3H), 1.42 (t, 2H), 1.52 (s, 3H), 1.59(s,3H), 1.87 (q, 2H), 5.03 (t, 1H), 5.80 (dd, 1H), 5.97(d, 1H), 6.17 (d, 1H), 6.68 (d, 2H), 7.16(d, 2H). | 154.90, 146.02, 135.66, 131.35, 130.62, 127.37, 126.58, 124.87, 115.50, 111.92, 42.55, 41.32, 25.77, 23.37, 23.27, 17.70. | Oily | 1.19(s,3H),1.46  (t,2H),1.52(s,3H),  1.63(s,3H),1.96(q,2H),  5.10(t,1H),5.89(q,1H),  6.05(d,1H),6.20(d,1H),  6.70(d,2H),7.20(2H),  9.37(s, 1H) | 156.7, 145.9, 134.0, 130.5, 128.4, 127.2, 126.6, 124.8, 115.4, 111.82, 42.2, 41.08, 40.53, 25.61, 23.03, 17.61 |
| Isopsoralen | 6.39(d, 1H), 7.14(m, 1H), 7.38, d, 1H), 7.44(d,1H), 7.70(m, 1H), 7.82(d, 1H) | 160.91, 157.35, 148.49, 145.91, 144.58, 123.84, 116.93, 114.12, 113.52, 108.84, 104.13. | White Crystalline  137-138 ^o^C (lit. value 137-138 ^o^C) | 6.40(d,1H),7.14(m,1H),  7.38(d,1H),7.44(d,1H),  7.70(m,1H),7. 82(d,1H) | 160.8,114.1,144.4,  123.8,108.8,157.4,  116.9,148.5,113.5,  145.8,104.1 |
| Psoralen | 6.39(d,1H), 6.84(d,1H), 7.49(s, 1H), 7.69(s,1H), 7.70 (d, 1H), 7.80(d, 1H) | 161.22, 156.43, 152.01, 146.96, 144.22, 124.93, 119.89, 115.42, 114.63, 106.40, 99.91. | White Solid  163-164 ^o^C (Lit. value 162-163 ^o^C) | 6.36(d,1H),6.84(d,1H),  7.48(s,1H),7.68(s,1H),  7.70(d,1H),7.80(d, 1H) | 160.8,114.5,144.0,  119.8,124.8,156.3,  99.6,151.9,115.3,  106.3,146.8 |
